# Gastrointestinal Bleeding in Patients with SARS-CoV-2 Infection Managed by Interventional Radiology

**DOI:** 10.3390/jcm10204758

**Published:** 2021-10-17

**Authors:** Anna Maria Ierardi, Andrea Coppola, Silvia Tortora, Elena Valconi, Filippo Piacentino, Federico Fontana, Elvira Stellato, Chiara Beatrice Cogliati, Daniela Torzillo, Emanuela Giampalma, Matteo Renzulli, Irene Bargellini, Roberto Cioni, Rossella Scandiffio, Angelo Spinazzola, Riccardo Alessandro Foà, Costantino Del Giudice, Massimo Venturini, Gianpaolo Carrafiello

**Affiliations:** 1 Radiology Unit, Fondazione IRCCS Cà Granda, Ospedale Maggiore Policlinico, Via Francesco Sforza 35, 20122 Milan, Italy; gcarraf@gmail.com; 2 Radiology Unit, Ospedale di Circolo e Fondazione Macchi, University of Insubria, 21100 Varese, Italy; coppola.andrea.mg@gmail.com (A.C.); f.piacentino@live.it (F.P.); fede.fontana@libero.it (F.F.); massimo.venturini@uninsubria.it (M.V.); 3 Postgraduate School of Radiology, University of Milan, 20122 Milan, Italy; silvia.tortora90@gmail.com (S.T.); elena.valconi@hotmail.it (E.V.); elvira.stellato@gmail.com (E.S.); 4 Department of Biomedical and Clinical Sciences “L. Sacco”, University of Milan, 20157 Milan, Italy; chiaracogliati@fastwebnet.it (C.B.C.); daniela.torzillo@fastwebnet.it (D.T.); 5 Radiology Unit, M. Bufalini Hospital, AUSL Romagna, 47023 Cesena, Italy; emanuela.giampalma@auslromagna.it; 6 Department of Radiology, IRCCS Azienda Ospedaliero-Universitaria di Bologna, 40133 Bologna, Italy; dr.matteo.renzulli@gmail.com; 7 Division of Interventional Radiology, Azienda Ospedaliero Universitaria Pisana, 56126 Pisa, Italy; irenebargellini@hotmail.com (I.B.); r.cioni2@gmail.com (R.C.); rossellascandiffio@libero.it (R.S.); 8 Unit of Interventional Radiology, Unit of Radiology, ASST Crema-Ospedale Maggiore, 26013 Crema, Italy; aspina@libero.it (A.S.); riccardo.foa@gmail.com (R.A.F.); 9 Department of Radiology, Interventional Radiology, Institut Mutualiste Montsouris, 75014 Paris, France; costantino.delgiudice@gmail.com

**Keywords:** COVID-19, gastrointestinal bleeding, GIB, risk factors, embolization, interventional radiology

## Abstract

Background: This study was conducted to evaluate the technical and clinical success of trans-arterial embolization (TAE) as a treatment of gastrointestinal bleeding (GIB) in Coronavirus Disease 2019 (COVID-19) patients and to describe its safety; moreover, we describe the characteristics of these patients. Methods: Thirty-four COVID-19 hospitalized patients presented with GIB. Risk factors, drugs administered for COVID-19 infection, and clinical and biological parameters were evaluated. Furthermore, intraprocedural data and outcomes of embolization were analyzed. Results: GIB was more frequent in male. Overweight, hypertension, diabetes, previous cardiac disease, and anticoagulation preadmission (48.5%) were frequently found in our population. Previous or actual COVID Acute respiratory distress syndrome (ARDS) and a high level of D-dimer were encountered in most cases. Upper GIB was more frequent than lower GIB. Technical and clinical success rates of embolization were 88.2% and 94.1%, respectively. The complication rate was 5.9%. Conclusions: Our study highlights the most frequent characteristics of COVID-19 patients with GIB. Embolization is feasible, effective, and safe.

## 1. Introduction

Coronavirus disease 2019 (COVID-19) is caused by a transmissible respiratory virus (SARS-CoV-2), detected in China in December 2019 and declared an official pandemic by the World Health Organization (WHO) on 11 March 2020 [1]. At the time of writing, there have been approximately 177,108,695 diagnosed infections and 3,840,223 deaths [1]. The clinical spectrum is wide, ranging from asymptomatic infection to severe viral pneumonia with respiratory failure, systemic involvement, and death [2,3,4]. Patients may have an increased susceptibility to develop coagulopathy, resulting in thromboembolism and disseminated intravascular coagulation (DIC) [5,6,7].

Recent studies have shown that COVID-19 patients treated with antithrombotic drugs are at increased risk of bleeding [8]. Among other mechanisms related to GI bleeding, stress ulcer formation from hospitalization [9] and hemorrhagic colitis possibly secondary to SARS-CoV-2 [10] have been mentioned.

Common GI symptoms include abdominal pain, nausea, vomiting, diarrhea [2,3,11], and sporadic gastrointestinal bleeding (GIB). Etiology is multifactorial, but not yet fully understood [2,3,11,12,13]. The rate of GIB events ranges between 1.5% and 13% [2,14].

The first-line treatment for GIB is endoscopy; however, this exposes staff to an increased risk of aerosol transmission of the virus [13] and patients to an increased risk of respiratory worsening during the procedure, with a possible need for respiratory support and transfer to the intensive care unit, often already saturated [4]. The risks of staff exposure need to be weighed against the benefits of endoscopy on a case-by-case basis using clinical judgement. Decisions could be better made using prognostic tools such as the Glasgow Blatchford score for the pre-endoscopic risk stratification of patients [15]. Moreover, recent studies have shown that some upper GIB can possibly be managed conservatively without endoscopy as patients responded within 24 h [16].

In COVID-19 patients, to avoid the aforementioned problems and in cases of persistent bleeding after endoscopy and preexisting hemodynamic instability, interventional radiology could play an important role [17,18].

The aim of our study was to describe the characteristics of patients with GIB and to evaluate the technical and clinical success, as well as the safety profile, of trans-arterial embolization (TAE) in the treatment of these hemorrhagic emergencies.

## 2. Materials and Methods

This was a multicenter retrospective observational study including 34 COVID-19 patients admitted to hospital between January 2020 and March 2021 with acute respiratory symptoms who developed GIB during hospitalization.

The study was approved by the ethics committee of the Coordinator Center (RadCovid 05-2020-467-2020).

We included all adult patients for a total of 34 patients (M:F, 22:12) hospitalized after a positive test by real-time polymerase chain reaction for COVID-19 infection. As mentioned above, all patients developed GIB during hospitalization. GIB was defined as evidence of hematemesis, coffee-ground emesis, melena, maroon stools, hematochezia, or a hemoglobin drop by 2 g·dL^−1^ in a 24 h period, decreasing in systolic blood pressure or hemodynamic instability.

Risk factors were assessed for each patient, especially body mass index (BMI) and comorbidities (hypertension, diabetes, cancer, cardiac disease, cirrhosis, etc.), as presented in Table 1. Moreover, drugs used for COVID-19 infection were reported, including prophylactic heparin (Table 2). More relevant clinical data and biologic parameters at hospital entry and the day of embolization were studied (Table 3 and Table 4). Moreover, intraprocedural data (Table 5) and outcomes of the embolization are reported (Table 6).

Most patients were evaluated by an emergency angio-CT to investigate the presence of active bleeding.

Indication for the procedure of embolization was established after a multidisciplinary consensus among gastroenterologist, radiologist, and surgeon on the basis of CT and/or endoscopic findings in association with clinical and laboratory data. In some cases, embolization was proposed after an unconclusive endoscopy or when endoscopy was not feasible [19,20].

Staff involved had to follow a high-standard infection protection protocol during the procedures [21].

Technical success was defined as the disappearance of contrast extravasation on post-procedural angiography or the completion of embolization of the desired artery (when an extravasation was not observed), while clinical success was established as the achievement of hemostasis, associated with hemodynamic stability, with no signs of rebleeding or related mortality within 30 days of embolization.

During follow-up, we monitored all patients’ symptoms and laboratory data every 6 h in the first 48 h and 1 week after the endovascular procedure.

Re-embolization was considered when clinical stability was not achieved during follow-up and/or evidence of persistent or new GI bleeding was demonstrated on a new angio-CT.

Safety was defined as procedure related morbidity and was evaluated according to the Society of Interventional Radiology guidelines [22].

All procedures were performed in the AngioSuite equipped for the treatment of COVID-19 patients, under local anesthesia, sedation, or general anesthesia with anesthesiologic assistance.

Access for endovascular angiography is usually gained via the common femoral artery; angiography is able to identify vessel(s) responsible for bleeding, and selective catheterization is carried out to prepare for embolization. In all hospitals, dedicated devices were used.

Given the small sample size and the observational nature of the study, only descriptive statistics were obtained for all variables assessed in the study population. Mean and standard deviation (SD) are provided for normally distributed variables, median and interquartile range (IQR) are provided for non-normally distributed variables, and number and percentage are provided for categorical variables. Normality was assessed by the Shapiro-Wilk test.

## 3. Results

Our results are only descriptive due to the small sample size.

In the series presented, GIB was more frequent in male (64.7%) with a median age of 71 years old.

Some risk factors were observed in patients with GIB; most of our patients were overweight (median BMI 29.52). Hypertension (82.4%), diabetes (29.4%), previous cardiac disease (52.9%), and anticoagulation preadmission (48.5%) were found more frequently in our patients. Among clinic parameters, previous or actual COVID ARDS was encountered in most of our cases (55.9%). Moreover, antiplatelet therapy before bleeding was registered in 32.3% of cases.

Almost all patients were treated with heparin as prophylactic therapy for COVID-19 infection.

D-dimer at hospital entry was high in most patients (median 865.5 (range 0.44–2680)).

Upper GIB was more frequent than lower GIB (64.7% versus 35.3%). Angiographic bleeding was encountered in 85.3% of our patients; in the remaining cases, the bleeding source was revealed by endoscopy but not successfully treated. Coils (52.9%) and onyx (32.4%) were the embolic agents more frequently used.

Technical and clinical success rates were 88.2% and 94.1%, respectively (Table 6). Five patients died (14.7%); in only one of these, death was related to hemorrhage; re-embolization was requested in 8.8% of the cases with resolution of hemorrhagic complication. Two complications (5.9%) related to the embolization procedure were registered: ischemic ulceration of the rectal mucosa and a migration of a millimetric glue emboli; in both cases, no consequences for patients were encountered.

As stated above, the small sample size may be considered the major limitation of our study; other important bias in our results may be related to the heterogeneity of our series collected in a multicenter fashion. On the other hand, our series of GIB in COVID-19 patients represents one of the most numerous in the literature, and multicenter collection may be the only way to get closer to a valid sample.

## 4. Discussion

COVID-19 is primarily a respiratory disease that causes a wide spectrum of clinical symptoms ranging from asymptomatic infection to acute respiratory distress syndrome (ARDS) and death [5,6,7].

Among extra-respiratory symptoms, we have identified gastrointestinal manifestations such as abdominal pain, nausea, vomiting, diarrhea, and gastrointestinal bleeding, the latter occurring at a rate ranging from 1.5% to 13% [2,14].

The etiology of gastrointestinal bleeding is multifactorial and not entirely clear. First of all, epithelial cells express the angiotensin 2-converting enzyme (ACE2), which represents an entry receptor of the virus, probably causing direct damage that leads to the formation of ulcers and bleeding [3]. The blockade of the ACE2 receptor, in close proximity to the amino-acid transporter B0AT, leads to a malfunction of the transport of amino acids with consequent reduced production of antimicrobial peptides, which leads to alteration of the intestinal microbiota and onset of enteritis [21].

Other factors that can contribute to gastrointestinal epithelial damage and bleeding are hospital-related stress ulcer formation, cytokine storm, disseminated intravascular coagulation, endorectal catheter, ECMO, anticoagulation, and antiplatelet therapy [23,24,25], although the latter does not appear to represent a statistically significant risk factor for bleeding [2,25]. Thromboprophylaxis plays a key role in the management of COVID-19 patients and requires routine evaluation of patients’ clinical-laboratory parameters to assess their bleeding risk.

In our study, almost all patients were treated with heparin as prophylactic therapy, and 32.3% of them were in antiplatelet therapy before bleeding. GIB during hospitalization represents a risk factor for mortality [25].

The current effective endoscopic and radiologic tools in managing an active GI bleeding should not be discouraged.

According to the literature [2,25], this study confirms that GIBs occur more frequently in the upper gastrointestinal tract (64.7% vs. 35.3%).

Some risk factors were observed in patients with GIB such as overweight (median BMI 29.52), hypertension (82.4%), diabetes (29.4%), previous cardiac disease (52.9%), anticoagulation preadmission (48.5%), and a high level of D-dimer at hospital entry (median 865.5).

The COVID-19 pandemic represents a challenge for medical care and hospital infrastructure worldwide, and new restrictions and recommendations are necessary in various healthcare settings, including endoscopy units, because of the potential for aerosol spread.

The first-line treatment for gastrointestinal bleeding is endoscopy; however, this exposes staff to an increased risk of aerosol transmission of the virus [13] and patients to an increased risk of respiratory worsening during the procedure, with a possible need for respiratory support and transfer to the intensive care unit, often already saturated [4].

In this study, we demonstrated that TAE in the management of GI bleeding is feasible, safe, and effective even in patients with COVID-19 infection, with technical and clinical success rates of 88.2% and 94.1%. Only one death was related to hemorrhage. Re-embolization was requested in 8.8% of the cases with complete resolution of hemorrhagic complication. Two complications (5.9%) related to embolization were registered without any consequences for patients.

Our study had several limitations. Firstly, the sample size was small, even though the number of COVID-19 patients collected that developed GIB during hospitalization represents one of the most numerous in the literature. As the COVID-19 pandemic spreads, we are looking to enroll a number of patients with GIB in new studies. Secondly, as a multicenter study, we are aware of the existing bias related to the heterogeneity of our series and data collection. As a descriptive study, we also know that we do not have statistically significant data that evaluate potential GIB risk factors, due to the small population included. The data collected permit only a preliminary assessment of the role of embolization in the management of this particular type of patient.

Furthermore, it would have been interesting to compare the incidence of GIB in a control group of non-COVID-19 patients or of non-hemorrhagic COVID-19 patients. Indeed, these should be topics for future evaluations.

## 5. Conclusions

In conclusion, COVID-19 patients deal with thrombosis and bleeding risks; this provides new and unique challenges in the emergency setting for whoever deals with these patients. When possible, a conservative approach with optimization of medical therapy is essential, but recourse to endoscopy and/or embolization techniques should be considered. Embolization may be more indicated in patients with a high risk of worsening respiratory function, as this approach has been well established to be safe for both patients and healthcare workers. Furthermore, our study highlighted the most frequent characteristics of COVID-19 patients with GIB.

## Figures and Tables

**Table 1:** Risk factors and descriptive statistics.

Risk Factors						
**Variables**	*n*	ND	Frequency (%)	Mean ± SD	Median (IQR)	Range
Sex Female Male	34	-	12 (35.3%) 22 (64.7%)	-	-	-
Age (years)	34	no	-	-	71 (63.75–79)	19–90
BMI	34	no	-	-	29.52 (24.85–33.63)	20.0–51.9
Weight (kg)	34	no	-	-	86.5 (73–100)	50–150
Height (m)	34	yes	-	1.71 ± 0.09	-	1.5–1.86
Asthma	34	-	1 (2.9%)	-	-	-
Hypertension	34	-	28 (82.4%)	-	-	-
Diabetes	34	-	10 (29.4%)	-	-	-
Cancer	34	-	7 (20.6%)	-	-	-
BPCO	34	-	4 (11.8%)	-	-	-
Renal failure	34	-	8 (23.5%)	-	-	-
Dialysis	34	-	0 (0%)	-	-	-
Immunodeficiency	34	-	5 (14.7%)	-	-	-
Active smoke	32	-	5 (15.6%)	-	-	-
Previous stroke	34	-	3 (8.8%)	-	-	-
Previous cardiac disease	34	-	18 (52.9%)	-	-	-
Previous pulmonary embolism	34	-	2 (5.9%)	-	-	-
Sleep apnea	34	-	3 (8.8%)	-	-	-
Previous hemorrhagic event	34	-	2 (5.9%)	-	-	-
Cirrhosis	34	-	0 (0%)	-	-	-
Treatment		-	-			-
Blood thinner preadmission ASA OAT Heparin	33	-	16 (48.5%) 9 (27.3%) 5 (15.2%) 5 (15.2%)	-	-	-
Hypertension treatment ACEi/sartan Loop diuretic Thiazide diuretic A-blocker B-blocker Ca^+^ channel blocker	34	-	20 (58.8%) 14 (41.2%) 4 (11.8%) 3 (8.8%) 4 (11.8%) 6 (17.6%) 8 (23.5%)	-	-	-
Diabetic treatment	34	-	3 (8.8%)	-	-	-

*n*: number of subjects; ND: normal distribution according to Shapiro-Wilk test; SD: standard deviation; IQR: interquartile range; BMI: body mass index; BPCO: Chronic Obstructive Pulmonary Disease; OAT: oral anticoagulant; ASA: acetylsalicylic acid; ACE: angiotensin I-converting enzyme.

**Table 2:** COVID treatment and descriptive statistics.

COVID Treatment						
Variable	*n*	ND	Frequency (%)	Mean ± SD	Median (IQR)	Range
Antiviral (darunavir; lopinavir–ritonavir + interferon; remdesivir; tocilizumab)	34	-	5 (14.7%)	-	-	-
Azithromycin	34	-	10 (29.4%)	-	-	-
Hydroxychloroquine	34	-	11 (32.4%)	-	-	-
Heparin	32	-	30 (93.8%)	-	-	-

*n*: number of subjects; ND: normal distribution according to Shapiro-Wilk test; SD: standard deviation; IQR: interquartile range.

**Table 3:** Clinic parameters and descriptive statistics.

Clinic						
Variables	*n*	ND	Frequency (%)	Mean ± SD	Median (IQR)	Range
Hospital entry → ICU admission (days)	28	no	-	-	5 (3.25–9)	0–24
Symptoms → bleeding (days)	22	no	-	-	10.5 (6.5–15)	4–36
ICU admission → bleeding (days)	4	yes	-	16 ± 6.48	-	10–23
Antiplatelet therapy before bleeding	31	-	10 (32.3%)	-	-	-
Tranexamic acid before bleeding	33	-	2 (6.1%)	-	-	-
Anti-inflammatory before bleeding	27	-	4 (14.8%)	-	-	-
DIC (CIVD)	31	-	0 (0%)	-	-	-
Previous or actual COVID ARDS	34	-	19 (55.9%)	-	-	-
ECMO	34	-	1 (2.9%)	-	-	-
Continuous hemofiltration	34	-	2 (5.9%)	-	-	-
Highest SAP 24 h before bleeding	19	yes	-	137.21 ± 33.85	-	74–207
Invasive procedure before bleeding	31	-	6 (19.4%)	-	-	
Total RB transfusion	34	no	-	-	3 (2–6)	0–20
Total plasma transfusion	34	no	-	-	1 (0–2)	0–19
Total platelet transfusion	34	no	-	-	0 (0–0)	0–3

*n*: number of subjects; ND: normal distribution according to Shapiro-Wilk test; SD: standard deviation; IQR: interquartile range; ICU: intensive care unit; DIC: disseminated intravascular coagulation; ARDS: acute respiratory distress syndrome; ECMO: extracorporeal membrane oxygenation; SAP: systolic arterial pressure; RB: red blood.

**Table 4:** Biologic parameters and descriptive statistics.

Biology						
Variable	*n*	ND	Frequency (%)	Mean ± SD	Median (IQR)	Range
D-dimer, hospital entry	34	no	-	-	865.5 (437.3–1273.3)	0.44–2680
Creatinine, hospital entry	34	no	-	-	1.1 (0.9–1.23)	0.6–2.39
GFR, hospital entry	16	yes	-	55.61 ± 29.03	-	15–101
cRP, hospital entry	19	yes	-	91.49 ± 88.04	-	0.52–230
Fibrinogen, hospital entry	10	no	-	-	7.2 (6.75–424.25)	5–707
TP, hospital entry	17	no	-	-	76 (65–95)	7–103
TCA, hospital entry	10	no	-	-	1.12 (1.01–11.38)	0.92–52
INR, hospital entry	18	no	-	-	1.2 (1.09–1.45)	1–10
Hct, hospital entry	21	yes	-	34.13 ± 6.11	-	24.4–43.5
Hb, hospital entry	21	yes	-	11.18 ± 2.23	-	8.1–14.9
Platelet, hospital entry	22	yes	-	235.77 ± 93.74	-	93–461
Troponin, hospital entry	12	no	-	-	35 (12.43–63.75)	7–133
White blood cell, hospital entry	21	yes	-	8.52 ± 2.99	-	3.5–14.49
D-dimer, ICU entry	10	yes	-	1454.84 ± 1129.37	-	1.47–2827
Creatinine, ICU entry	14	no	-	-	87.5 (78.25–149.25)	51–239
GFR, ICU entry	13	yes	-	71.54 ± 34.94	-	22–141
cRP, ICU entry	11	no	-	-	193 (140–229)	8.84–268
Fibrinogen, ICU entry	12	no	-	-	6.99 (6.3–8.13)	1–647
TP, ICU entry	13	yes	-	75.54 ± 22.41	-	34–103
TCA, ICU entry	12	no	-	-	1.25 (1–1.98)	0.92–62.4
INR, ICU entry	10	no	-	-	1.34 (1.1–2.35)	1–10
Hct, ICU entry	14	yes	-	33.02 ± 7.39	-	22–41.8
Hb, ICU entry	34	yes	-	11.11 ± 1.97	-	8–14.2
Platelet, ICU entry	34	yes	-	233.76 ± 85.29	-	93–461
Troponin, ICU entry	11	no	-	-	29 (16–55)	11.9–345
White blood cell, ICU entry	14	yes	-	8.82 ± 4.26	-	0.1–16.9
D-dimer, day emb	34	yes	-	644.21 ± 477.64	-	0.44–1654
Creatinine, day emb	34	no	-	-	1.15 (0.9–1.39)	0.56–98
GFR, day emb	21	yes	-	54.81 ± 31.25	-	13–125
cRP, day emb	17	no	-	-	8.56 (2.32–23.53)	0.5–135
Fibrinogen, day emb	14	no	-	-	4.4 (3.63–158.75)	1–864
Anti-Xa, day emb	9	no	-	-	0.26 (0.1–0.88)	0.07–2.04
TP, day emb	20	yes	-	71.75 ± 15.07	-	35–91
TCA, day emb	13	no	-	-	1.13 (0.97–12.92)	0.9–36.4
INR, day emb	34	no	-	-	1.3 (1.1–1.44)	1–2.5
Hct, day emb	34	no	-	-	23.6 (21.78–28.88)	15.9–39
Hb, day emb	34	no	-	-	7.6 (7.1–8.43)	5.4–11.1
Platelet, day emb	34	no	-	-	184 (148–283.25)	63–432
White blood cell, day emb	34	no	-	-	13.18 (10.05–18.58)	4.13–40.5

*n*: number of subjects; ND: normal distribution according to Shapiro-Wilk test; SD: standard deviation; IQR: interquartile range; GFR: glomerular filtration rate; INR: international normalized ratio; Hb: hemoglobin; GFR: glomerular filtration rate; cRP: C-reactive protein; TP: total protein; TCA: tricyclic antidepressants; Hct: hematocrit.

**Table 5:** Intervention and descriptive statistics.

Intervention						
Variable	*n*	ND	Frequency (%)	Mean ± SD	Median (IQR)	Range
Bleeding Site Lower GI Upper GI	34	-	12 (35.3%) 22 (64.7%)	-	-	-
Time Ct scan → embolization (hours)	34	no	-	-	3.5 (2–6)	1–24
Anesthesia Local Sedation General	34	-	10 (29.41%) 14 (41.17%) 10 (29.41%)	-	-	-
Angiographic bleeding	34	-	29 (85.3%)	-	-	
Number of embolized arteries	34	no		-	1 (1–1.25)	1–2
Embolizing agent Onyx Glue Coils	34	-	11 (32.4%) 7 (20.6%) 18 (52.9%)	-	-	-

*n*: number of subjects; ND: normal distribution according to Shapiro–Wilk test; SD: standard deviation; IQR: interquartile range.

**Table 6:** Outcomes and descriptive statistics.

Outcomes						
Variable	*n*	ND	Frequency (%)	Mean ± SD	Median (IQR)	Range
Need of re-embolization	34	-	3 (8.8%)	-	-	-
Clinical success	34	-	32 (94.1%)	-	-	-
Death	34	-	5 (14.7%)	-	-	-
Death related to bleeding	34	-	1 (2.9%)	-	-	-
Complication of embolization	34	-	2 (5.9%)	-	-	-
Hospital stay (days)	29	no	-	-	45 (39.5–65)	29–106

*n*: number of subjects; ND: normal distribution according to Shapiro–Wilk test; SD: standard deviation; IQR: interquartile range.

## Data Availability

Data reported in the present manuscript are available in each center which pertains to the multicenter study.
